# A Study on the Stability of Water-Gated Organic Field-Effect-Transistors Based on a Commercial p-Type Polymer

**DOI:** 10.3389/fchem.2019.00667

**Published:** 2019-10-10

**Authors:** Rosaria Anna Picca, Kyriaki Manoli, Eleonora Macchia, Angelo Tricase, Cinzia Di Franco, Gaetano Scamarcio, Nicola Cioffi, Luisa Torsi

**Affiliations:** ^1^Dipartimento di Chimica, Università degli Studi di Bari “Aldo Moro”, Bari, Italy; ^2^Consorzio per lo Sviluppo dei Sistemi a Grande Interfase, Unità di Bari, Bari, Italy; ^3^The Faculty of Science and Engineering, Center for Functional Materials, Åbo Akademi University, Turku, Finland; ^4^CNR - Istituto di Fotonica e Nanotecnologie, Unità di Bari, Bari, Italy; ^5^Dipartimento Interateneo di Fisica “M. Merlin”, Università degli Studi di Bari “Aldo Moro”, Bari, Italy

**Keywords:** poly-3-hexylthiophene, electrolyte-gated OFET, degradation, pulsed mode, biosensors

## Abstract

Robust electrolyte-gated organic field-effect-transistors (OFETs) are particularly needed for the development of biosensing devices. However, when a FET biosensor operates in aqueous environments or even in real biological fluids, some critical issues may arise due to the possible lack of environmental long-term and/or operational stability. An important source of instability is associated with the degradation of the organic electronic channel materials such as for instance, poly-3-hexylthiophene (P3HT), a benchmark commercially available p-type organic semiconductor. In this work, the investigation of critical parameters, such as the control over spurious electrochemical phenomena as well as the operating conditions that can affect water-gated OFETs lifetime, is reported, together with a proposed modeling of the P3HT stability curve over 1 week in water. The investigation of possible morphological/chemical modifications occurring at the polymer surface after operating in water for 2 weeks was carried out. Moreover, it is proven how the addition of a gel layer can extend the P3HT based water-gated OFET shelf life up to 2 months.

## Introduction

The use of organic field-effect-transistors (OFETs) as sensors (Torsi et al., 2013; Li et al., 2018; Surya et al., 2019) has received a tremendous boost in the last decades thanks to the advent of low-cost fabrication strategies as well as of flexible and/or stretchable substrates (Kim et al., 2013; Manoli et al., 2015). However, their exploitation and commercialization as real-life sensors have been limited by the organic polymer degradation occurring when the device is operated in aqueous environments (Knopfmacher et al., 2014; Wang et al., 2016; Zhang et al., 2016). Extensive literature on the operational and environmental stability of organic semiconductors have been reported for devices working with solid-state dielectrics (Salleo et al., 2005; Sirringhaus, 2009; Bobbert et al., 2012; Nikolka et al., 2016; Lee et al., 2017; Jia et al., 2018) addressing the role of external agents (e.g., water, oxygen, light) in reducing the device performance. Evidences of device degradation encompass the increased hysteresis on transfer curves and leakage (I_G_) for successive measurements, threshold voltage (V_T_) shift, on current (I_on_) decrease with time (Sirringhaus, 2009). Such behavior has been mainly ascribed to the uncompensated charged trapped in deep localized states in the semiconductor also caused by spurious electrochemical processes (Sirringhaus, 2005; Beatrup et al., 2014; Porrazzo et al., 2014). For example, the presence of polaronic states can cause an increased susceptibility to the electrochemical degradation (Wade et al., 2015). In fact, the presence of hole polarons under device operation is found to make polaron degradation pathways accessible (Beatrup et al., 2014). Moreover, a charge transfer complex may be formed with oxygen (favored under light) leading to instability (Bellani et al., 2014).

Some alternatives to overcome such drawbacks have been proposed, such as the inclusion of ZnO nanophases in the bioreceptor layer of OFET biosensors (Picca et al., 2018) or polymer encapsulation (Yu et al., 2016; Lee et al., 2017). More recently, electrolyte-gated devices (EGOFETs) have emerged as interesting devices that can operate in the sub-volt regime (Panzer and Frisbie, 2008; Kergoat et al., 2010; Cramer et al., 2013) thus paving the way to their application in label-free ultra-sensitive biosensors (Mulla et al., 2015; Macchia et al., 2018a,b, 2019a,b).

In EGOFETs, two electrical-charge-double-layers (EDL) are built: one at the gate/electrolyte and the other at the electrolyte/organic semiconductor interfaces. When water serves as electrolyte, the EDL capacitance is remarkably high, being in the order of few μF^*^cm^−2^ (Cramer et al., 2013). In such an architecture, the direct contact between the organic semiconductor (OS) layer and the aqueous environment rises concerns about the already investigated degradation induced in such polymers by electrochemical processes that occur in water (de Leeuw et al., 1997; Sharma et al., 2010; Bobbert et al., 2012), that are particularly detrimental leading to unstable EGOFETs on prolonged use (Cramer et al., 2013; Bellani et al., 2014; Porrazzo et al., 2014). Until now, only few studies about EGOFET devices have addressed this issue reporting on stability over time (Zhang et al., 2016) or under harsh environments (Porrazzo et al., 2014; Algarni et al., 2016). In this work, poly-3-hexylthiophene (P3HT) was chosen as benchmark p-type OS used to fabricate water-gated OFETs, employing a bulk gold foil as gate electrode. At first, the reduction of hysteretic behavior was demonstrated by setting up a suitable measuring protocol. Moreover, environmental (incubation in HPLC water) and operational stability (cycling mode) studies were carried out demonstrating the main role of the electrolyte over the measuring conditions in device degradation. A suitable cell was also designed to overcome the problem of water evaporation during measurements. Interestingly, P3HT degradation was modeled in terms of I_D_ decrease with a two-phase exponential decay function indicating that a first process occurs within few hours, whereas a second one has a time constant in the order of 35 h. It was demonstrated that a similar trend is mainly associated to V_T_ negative shift with time. A strategy to extend EGOFET use over the months was also investigated. Additional morphological and spectroscopic characterizations performed on as-prepared and used devices provided information about the response of P3HT film to water contact on a long time scale (2 weeks).

## Materials and Methods

### Fabrication of EGOFET Devices

Highly n-doped silicon substrates, covered by 300-nm thermally grown SiO_2_, were purchased from Si-Mat®. Source (S) and drain (D) interdigitated electrodes were photo-lithographically patterned on the Si/SiO_2_ wafer. To this end, a Ti (99.995%) film (5 nm thick) was deposited as adhesion layer prior of the Au (99.99%) film (50 nm thick) deposition by electron-beam evaporation. The S&D interdigitated electrodes have a channel length (L) of 5 μm and a channel width (W) of 10,560 μm. Poly(3-hexylthiophene-2,5-diyl) (regioregular, electronic grade, 99.995% trace metal basis, average M_n_ ~17,500, Sigma-Aldrich), P3HT, was used as p-type OS throughout the study. The polymer was first dissolved in chlorobenzene (2.6 mg/mL), and then the solution was sonicated for 20 min prior to filtration through a PTFE 0.2 μm syringe filter. Before depositing the OS film, substrates bearing the interdigitated electrodes were cleaned under ultrasounds in an acetone bath (10′) followed by a similar step in 2-propanol and finally dried under nitrogen. P3HT film was spin coated at 2,000 rpm for 20 s and annealed at 90°C for 10 min. A channel area of 6.4·10^−3^ cm^2^ was estimated (Macchia et al., 2018a). The gate electrode (G) consisted of an Au bulk sheet (total area ~1.5 cm^2^) mounted in L-configuration, whose water immersed area was about 0.6 cm^2^. When specified, a channel consisting of P3HT/ZnO nanoparticles hybrid layer was prepared by spin coating starting from a filtered P3HT solution mixed with ZnO nanoparticles (Picca et al., 2015) (1.5 mg/mL final concentration).

**Figure 1:**
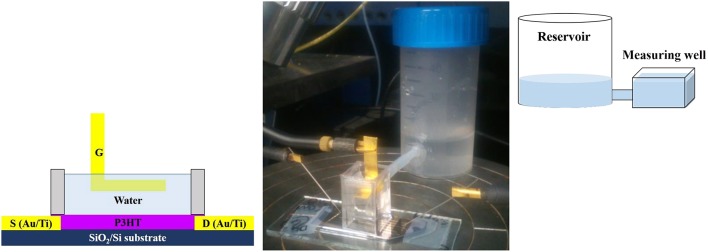
Scheme and picture of the set up used for studying EGOFET devices. A top-gate bottom-contacts configuration is adopted.

A suitable well for performing long-term studies under water was designed and fabricated to prevent possible water evaporation. It consists of a polystyrene (PS) cell glued with polydimethylsiloxane on the substrate. A PTFE tube connects the cell to a proper water reservoir in order to keep the water level constant in the well. The well is filled with 1.2 mL of water (HPLC-grade, Honeywell Riedel-de Haën) acting as gating medium. The scheme and the picture of the measuring set up are presented in Figure 1.

### EGOFET Electrical Characterization

All the devices were characterized by means of a Keithley 4200-SCS semiconductor characterization system in air at room temperature (20–22°C) in a dark box. The drain current (I_D_) was acquired scanning the gate voltage (V_G_) between 0.1 and −0.5 V, keeping the drain voltage (V_D_) at −0.4 V. The voltage step was set at 0.01 V. The source is grounded as typical in common source configuration measurements. Forward and backward scans were performed to evidence the occurrence of hysteretic behavior. A suitable measuring protocol was also set up as described in the results section. The potential window was defined according to the need to prevent any electrochemical process to occur and affect the EGOFET. To this aim, gate current (I_G_) was also registered in order to monitor any spurious electrochemical process. Electrical figures-of-merit (μ_FET_ and V_T_) were extracted from the I_D_-V_G_ transfer characteristics in the saturation regime according to a well-known procedure (Torsi and Dodabalapur, 2005) and are averaged on 10 different devices (*n* = 10) operated in independent measurements. Error bars refer to one standard deviation calculated considering *n* = 10.

### Morphological Analysis

**Figure 2:**
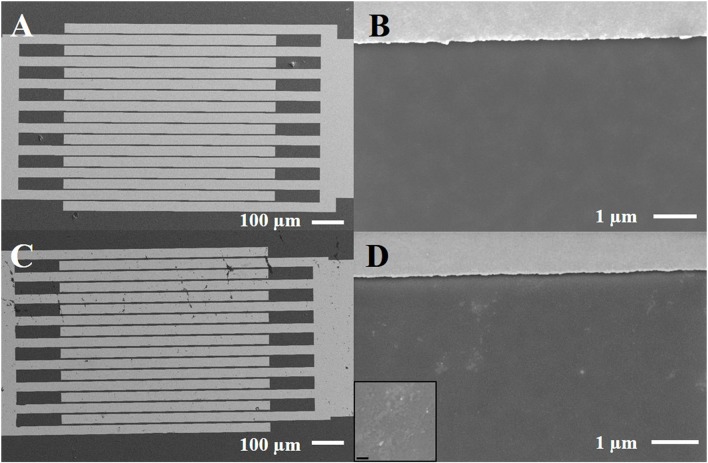
Scanning electron micrographs of P3HT film deposited onto interdigitated electrodes, before **(A,B)** and after 2 weeks under water **(C,D)**. The inset refers to a zoom at higher magnification (scale bar corresponds to 200 nm) of the exposed sample.

Scanning electron microscopy (SEM) was applied to investigate the in-plane homogeneity of OS films deposited by spin coating. Analysis was performed by means of a Carl Zeiss Σigma field emission SEM on films prepared, both on interdigitated electrodes and on SiO_2_/Si substrates. The probing electron beam was set at an acceleration voltage of 3–5 kV acquiring images at different magnifications in top-view. SE2 type detector was used to acquire the overall image of the device (Figures 2A,C), whereas the detailed view of the samples was recorded with the in-lens detector (Figures 2B,D).

Atomic Force Microscopy (AFM) characterization was performed on P3HT-based EGOFETs, just after their preparation and after contact with water for 2 weeks. AFM images were collected with a NT-MDT mod. Ntegra microscope in semi-contact mode using a tip apex size of 10 nm at a frequency *f* = 180 kHz. For each sample, areas of 3 × 2 μm were investigated. The Image Analysis Software was used to evaluate the surface roughness as route mean squared (RMS) on at least three representative areas of the sample.

### X-ray Photoelectron Spectroscopy Surface Analysis

P3HT-based FETs were analyzed by means of X-ray photoelectron spectroscopy (XPS) using a PHI Versaprobe II Spectrometer. A monochromatized Al Kα radiation (1486.6 eV) was used. Survey spectra were acquired with a pass energy of 117.4 eV; whereas high-resolution (HR) spectra were acquired with a pass energy of 58.7 eV. C1s, O1s, Si2p, S2p, Au4f regions were investigated. Both as-prepared and used samples were characterized. MultiPak™ (v. 9.7.0.1, PHI-ULVAC) software was used to process the data. Binding energy (BE) scale was corrected taking as reference C1s component at 284.8 eV. Three representative areas of the sample were collected to evaluate the chemical surface composition.

## Results and Discussion

### SEM Characterization of P3HT Films

P3HT organic semiconductor has been typically successfully used by our group as active channel in FET biosensors with different architectures (Angione et al., 2012; Magliulo et al., 2013; Macchia et al., 2016). As a general procedure, P3HT films were prepared by spin-coating starting from a chloroform solution. However, SEM investigation on similar samples showed that P3HT layers were not uniform due to the presence of some voids and agglomerates (Sportelli et al., 2017). Chlorobenzene was then selected because of its higher boiling point as alternative solvent to improve layer uniformity and crystallinity, in agreement with what previously reported in the literature (Kergoat et al., 2011). SEM images of typical P3HT films, freshly deposited on a plain SiO_2_/Si substrate and on gold electrodes, are presented in Figures 2A,B. It is evident that a rather uniform OS layer is formed confirming the advantages of processing P3HT from chlorobenzene. The very same samples were put in contact with water for 2 weeks and used as EGOFET devices (*vide infra*) and then characterized by SEM to evaluate a possible modification of layer uniformity. Additional features appear in the images (Figures 2C,D), though it seems that they are mainly related to the effect of water evaporation and presence of dust/salt particles (see the inset of the Figure). In fact, cracks and/or delamination of the polymer are not evident.

### AFM Characterization of the OS Layer

**Figure 3:**
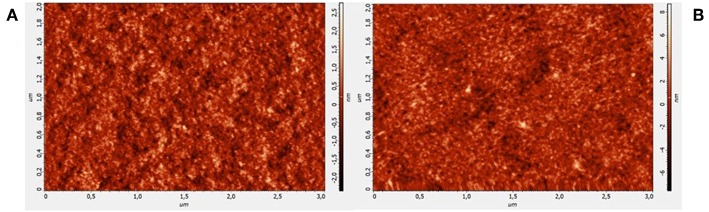
AFM images of P3HT film deposited onto interdigitated electrodes, before **(A)** and after 2 weeks under water **(B)**.

Figure 3 shows two typical AFM images of an area of a P3HT FET, before (a) and after (b) 2-week exposure to water. RMS was calculated as an estimate of the roughness in both cases. A general increase in the roughness going from 0.5 ± 0.1 nm for pristine samples to 1.6 ± 0.2 nm after use was found. The low roughness observed on freshly prepared films is indicative of the goodness of the solvent using for processing the polymer in agreement with SEM results. After 2-week contact with water, the RMS increment can be attributed to a sort of polymer swelling. Nonetheless, dramatic changes in the surface morphology are not visible which account for the preservation of the FET modulation, at least in the investigated time scale.

### XPS Characterization of P3HT Active Layers

**Figure 4:**
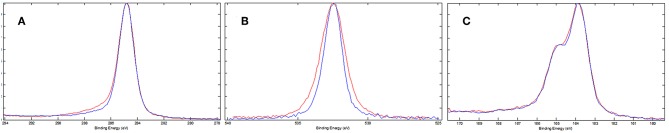
C1s **(A)**, O1s **(B)**, S2p **(C)** XP regions registered for P3HT-based FETs, before (blue curves) and after contact with water for 2 weeks (red curves).

The surface chemical composition of fresh and used P3HT-based devices was determined by XPS analysis (Table 1). The chemical composition does not seem particularly affected by contact with water, apart the higher oxygen content. Such finding is confirmed by spectral comparison of C1s, O1s, S2p regions (Figure 4) acquired before and after long exposure to water. Slight increment in carbon oxygenated components is observed (BE > 286 eV) suggesting a certain chemical degradation of the polymer. Nonetheless, the lineshape of S2p signal is preserved suggesting no oxidation on Sulfur. Interestingly, increment in the oxygen content is correlated to the presence of hydroxyl groups in addition to the O1s component ascribed to SiO_2_ (BE = 532.5 ± 0.2 eV) (National Institute of Standards and Technology, 2012). XPS results suggest then that a partial modification of the polymer occurs within the investigated period, accounting for the increased roughness and the reduced electrical performance upon long exposure to water.

**Table 1:** Typical surface chemical composition of fresh (*t* = 0) and aged (*t* = 15 days in water) P3HT samples.

**Sample ID**	**C%**	**S%**	**O%**	**Si%**	**Au%**
P3HT (*t* = 0 day)	79.6	5.7	9.1	5.3	0.3
P3HT (*t* = 15 days)	80.5	5.4	10.9	3.0	0.2

*Error on atomic percentages (At%) is ±0.2% for Au and ±0.5% for all the other elements*.

### The Measuring Protocol

**Figure 5:**
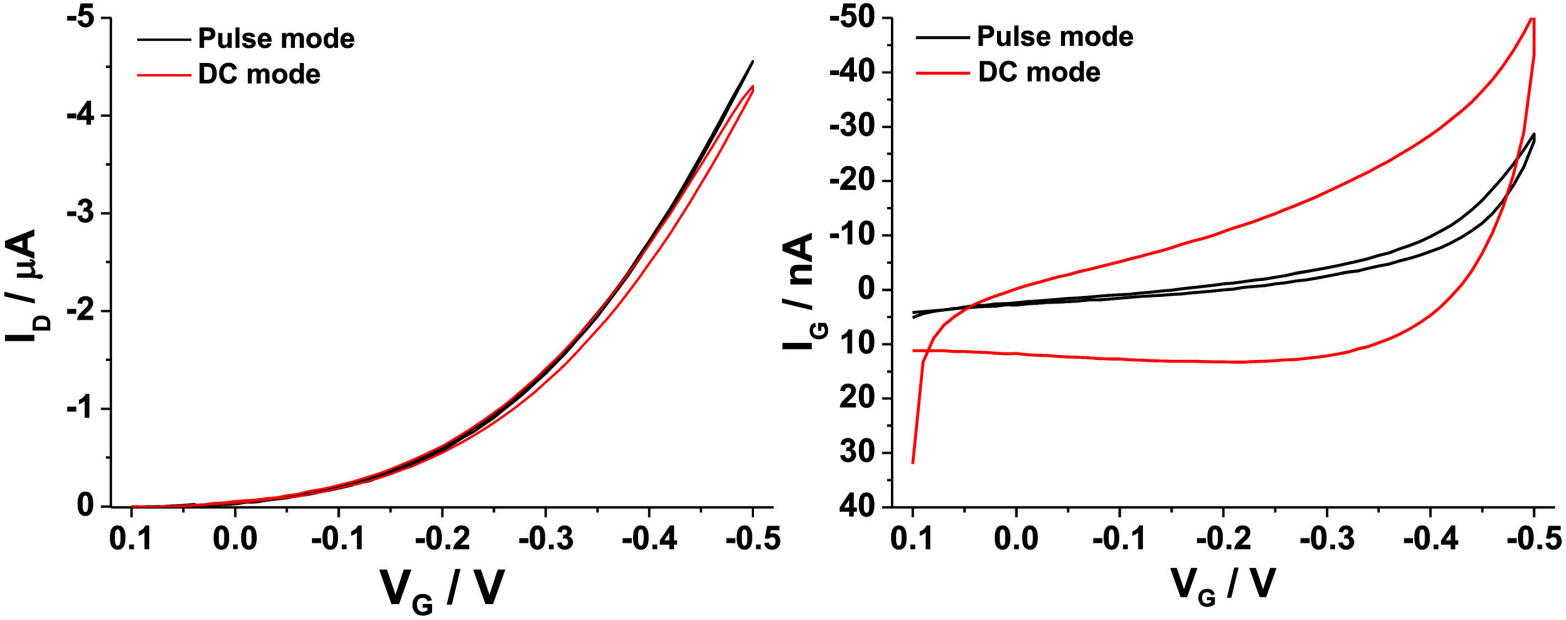
I_D_-V_G_ **(left)** and I_G_-V_G_ **(right)** curves registered on a P3HT water-gated FET in pulse (black curves) or continuous (red curves) mode.

As anticipated, hysteresis, i.e., the difference between I_D_ values measured in the forward and backward gate voltage sweep, is one of the main issues that can arise when OFET devices are operated. In these cases, this behavior has been often attributed to the presence of moisture in the OS layer or at the OS/dielectric interface, especially when SiO_2_ is used as gate dielectric (Bobbert et al., 2012). On the other hand, water-gated OFETs show minor hysteresis phenomena. This is mainly attributed to the absence of proton trapping in a dielectric and the occurrence of their neutralization by water autoprotolysis (Cramer et al., 2013). Hysteresis can be influenced by the sweeping potential window and by the scan rate of the applied potential (Panzer et al., 2005). For example, it was shown that scanning the V_G_ from the *off* to the *on* state (and *vice versa*) by applying pulses of alternating polarities was a suitable method to get hysteresis-free transfer curves in P3HT based FETs (Manoli et al., 2014). In this work, a similar protocol was adopted as reported in the Supplementary Material. It was observed that, regarding V_G_ sweep protocol, keeping the *on* time equal to 10 ms (+1 s cumulative measure time) as compared to the *off* time (1 s) and selecting −0.05 V as base voltage, gave the best results. This means that a slow scan rate of about 40 mV/s is applied to V_G_. Moreover, the V_D_ set at a maximum value of −0.4 V, was also pulsed keeping the *on* time (1 s) longer than the *off* time (0.1 s) at 0 V. A comparison between the performances of water-gated P3HT FETs operated in the DC-mode (red curves) or in the pulse-mode (black curves) is presented in Figure 5. The drain and the leakage currents (I_G_) are reported as a function of V_G_ for the two cases. Apparently, the pulse mode contributes to measure a slightly increase I_D_, and more relevantly to measure an almost hysteresis-free and reduced gate leakage current level. Taking the current values at V_G_ = −0.5 V, the ratio I_D_/I_G_ in the pulse-mode was about twice the one measured in the DC-mode.

### Operational Stability

**Figure 6:**
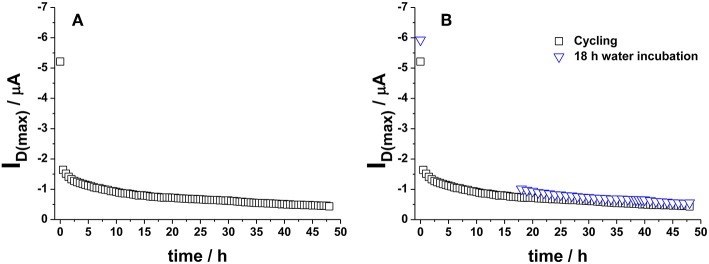
**(A)** I_D(max)_ (measured at V_G_ = −0.5 V) variation over 48 h when cycling mode is applied to water-gated P3HT FET. Data refer to transfer curves acquired every 30 min. **(B)** Comparison between I_D(max)_ values reported in **(A)** and I_D(max)_ registered on a water-gated P3HT FET incubated in water for 18 h (without operating it) (blue hollow triangles).

**Figure 7:**
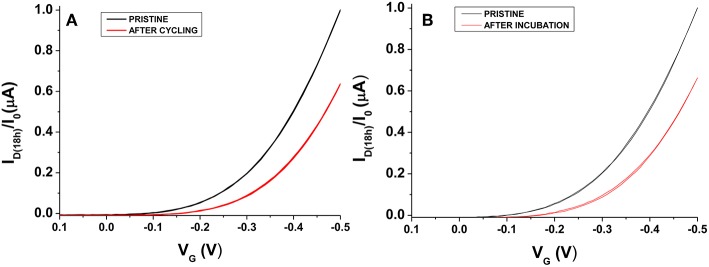
I_D_-V_G_ curves taken before (black) and after (red) 18 h for water-gated P3HT FETs operated in cycle mode **(A)** or just put in contact in water **(B)**. I_D_ has been normalized on the initial I_D_ value (I_0_).

The definition of a proper measurements protocol was beneficial to improve EGOFET performance level; however, it was not sufficient in limiting the degradation phenomena and hence the device long-term stability and reliability. In this respect, it is worth to dwell into the operational stability as well. A general scheme reported in literature involves the use of the so-called bias-stress mode consisting in the application of a continuous gate bias over time (Sirringhaus, 2009), though other approaches based on the cycling measurements of transfer curves have also been applied (Hwang et al., 2011). In this work, the interest was focused on the aspects connected with the application of EGOFETs as biosensing platforms; therefore, the operational stability of P3HT based devices was studied in water. To this aim, the typical sensing protocol followed in our laboratory in single-molecule detection (Macchia et al., 2018a) was simulated and the transfer curves of the fabricated devices were measured every half an hour using water and Au sheet as gating medium and gate electrode, respectively. A 30 min interval was chosen to mimic the time generally passing between two successive exposures to analyte solutions in a conventional sensing experiment. In fact, it is fundamental to get highly stable devices before using them in sensing platforms (Macchia et al., 2019b). P3HT based devices were then tested just after their fabrication. An example of the variation of the maximum current, I_D(max)_ (measured at V_G_ = −0.5 V), over 48 h is shown in Figure 6A. It can be seen that a dramatic decrease in current occurs in the first cycles, whereas the current level almost reaches a steady state after 24 h. This trend was not surprising considering that the devices are not operated in an inert atmosphere. An alternative approach was explored to discriminate both the role of the measuring environment and of the operational protocol. Some devices were kept in contact with water just after their preparation (pristine sample) without operating them for the first 18 h. In this case, I_D(max)_ variation over time (blue hollow triangles) is presented in Figure 6B. Interestingly, the two experiments led to very similar results, thus suggesting that the pronounced initial I_D_ decrease is essentially correlated to the adjustment of the polymer film in water environment. Quantitatively, a current decay of about 35% was generally observed for both the 18 h-cycled or the 18 h-water-incubated samples as reported in Figure 7. This fundamental aspect has never been clearly addressed in previous works about operational stability of OFET devices in water. It is worth noting that a similar decay was also reported for devices based on OS blended with polystyrene after 11 h of operation in water. In this case, a current decrease rate of about 3%/h was reported (Zhang et al., 2016). In this work, we observed that devices comprising a commercial P3HT seem more stable even in the first day after their preparation. After this period, the current drift is typically reduced and is around 1%/h allowing the P3HT based device to be very well suited to be used in ultra-sensitive biosensing experiments (Macchia et al., 2019b).

The timescale of this characterization was further extended taking into account the variation of the electrical figures-of-merit, μ_FET_, V_T_, and I_D(max)_, over a time lease of 2 weeks. In this case, the devices were kept in water and measured in cycling mode for 5 h per day. The current values at *t* = 0 day and at *t* = 15 days are given in Table 2. The EGOFET devices comprising a P3HT channel material modified with an n-type nanostructured oxide were also tested under the same conditions. Such investigation was undertaken because in our previous studies ZnO nanoparticles were implemented in OFETs giving good performance in terms of electrical properties (Picca et al., 2015) and stability (Picca et al., 2018).

**Table 2:** Device figures of merit estimated on fresh and aged samples (used for 2 weeks).

**Sample ID**	**μ_FET_ (cm^**2**^V^**−1**^s^**−1**^)**	**V_**T**_ (V)**	**|I_**D(max)**_| (μA)**
P3HT (*t* = 0 day)	(5 ± 2)*10^−2^	−0.03 ± 0.05	2.8 ± 1.2
P3HT (*t* = 15 days)	(4 ± 2)*10^−2^	−0.16 ± 0.05	1.1 ± 0.8
P3HT/ZnO (*t* = 0 day)	(7 ± 3)*10^−2^	−0.04 ± 0.03	5 ± 2
P3HT/ZnO (*t* = 15 days)	(7 ± 3)*10^−2^	−0.13 ± 0.03	3 ± 2

*A comparison with P3HT devices bearing ZnO nanoparticles (P3HT/ZnO) is shown. Errors refer to one standard deviation for n = 10*.

At a first inspection, it can be observed that the device degradation translates into a negative shift of the threshold voltage, V_T_. Interestingly, mobility seems almost unaffected by the prolonged device operation in water. Similar findings were reported in other systems, as well. Moreover, the electrical performance and the stability of the devices were not significantly improved by the addiction of the ZnO nanostructures. This is not so surprising since in the previous work ZnO was deposited as separate layer underneath the P3HT film in a functional bio-interlayer OFET biosensor, thus behaving as capturing layer for water molecules at the interface between P3HT and the solid dielectric (Picca et al., 2018). On the other hand, recent data on water-gated devices based on P3HT films modified with WS_2_ nanotubes showed an increased operational stability, quite likely due to the nanoscale morphology of the active blend layer (Macchia et al., 2018c).

**Figure 8:**
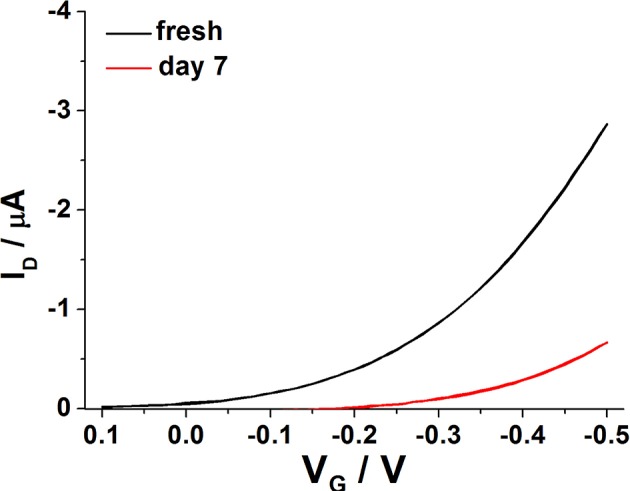
Typical transfer curves registered on a water-gated P3HT FET, just after fabrication (black line) and after 7 days (red line).

As expected, the degradation cannot be avoided but it is in the order of the one reported for EGOFETs based on other materials. In fact, the current level was reduced of about 30% of its original value after 7 days (Figure 8), in agreement with data available in literature (Zhang et al., 2016). Furthermore, the study was focused on the modeling of device degradation under the experimental conditions explored in this work. In the past, works related to devices operating with a solid-state dielectric under bias stress proved that the V_T_ shift correlates with current decay, whereas mobility is almost unchanged (Salleo et al., 2005). Bias stress instability translates into a voltage threshold shift, and hence into I_D_ decay, which can be generally modeled with a single exponential decay function (Zhang et al., 2009). In the paper by Salleo et al. (2005) it was also observed that on a very short timescale (<100 s) almost all the charged traps could be recovered after bias removal indicating that these defect states have a short lifetime and are probably located in the more ordered regions of the organic semiconductor. In the same work, a long-lived component of trapped charges was also invoked. These traps are characterized by slow kinetics as they can last for days, thus being responsible for the observed lower V_T_ shift. It is believed that they are present in deep states, quite likely distributed in the disordered portions of P3HT.

**Figure 9:**
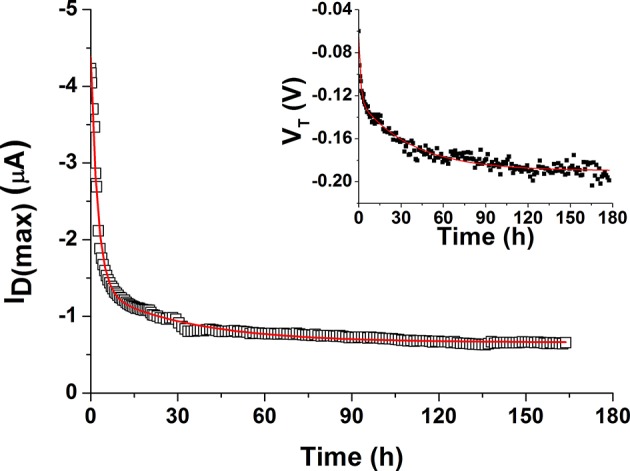
Modeling of P3HT degradation over 7 days, expressed as I_D(max)_ vs. time. In the inset, the shift in the threshold voltage is also fitted with the same function.

In the field of EGOFETs, little is known about device degradation under operation and empirical approaches are typically employed to describe their behavior, typically carrying out the studies on a limited period (hours). Based on the experimental conditions employed in this work, the degradation of the prepared devices was followed in terms of I_D(max)_ decay and V_T_ variation over 7 days (Figure 9). A double exponential decay function, expressed as y = A1^*^exp(–x/t1) + A2^*^exp(–x/t2) + y0, was used to fit both parameters. It is worth mentioning that such expression was already used in other works to describe recovery in pulse duty cycle studies (Miyadera et al., 2008; Manoli et al., 2014). However, both previous papers referred to OFET systems. The two extracted time constants (t1, t2) are similar for both drain current and threshold voltage changes, in agreement with previous observations. In particular, t1 is 1.8 ± 0.5 h and t2 is 35 ± 3 h. This means that two distinct processes impact on P3HT degradation processes occurring at different times. The first decay is responsible for the “rapid” current loss in the initial time-frame right after the incubation in water. Such trend could be in principle recovered within few days after stress removal (Sirringhaus, 2009). The second process may result from the presence of uncompensated immobile charges in the OS film. Obviously, due to continuous contact with the electrolyte, other effects such as the partial penetration of water in the P3HT film as well as formation of a passivating oxide layer on gold should be also taken into account. In particular, AFM and XPS results indicate that slight modifications in terms of morphology and composition of the polymer occur when devices are operated in water over a long period thus supporting the existence of degradation pathways responsible for the irreversible components of the decay model. It was generally reported that water molecules can penetrate the OS layer being then enclosed in the polymer nanovoids and causing trap formation (Nikolka et al., 2016; Zuo et al., 2019). This means that the latter component of degradation cannot be easily recovered and further studies will be necessary to clarify this behavior.

### Shelf Life Extension

**Figure 10:**
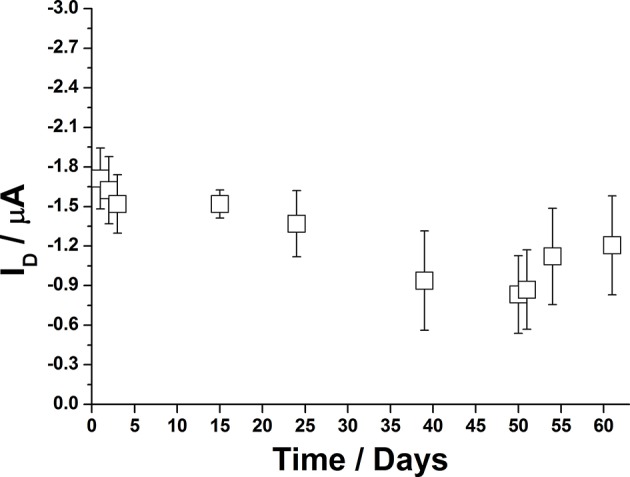
Long-term stability of P3HT based EGOFETs, stored with a protecting layer of agarose gel. Error bars refer to one standard deviation calculated on three devices.

An important aspect for the development of EGOFET biosensors is related to their storage as well as to the possibility to use them several times. When continuously operated in water, it was observed that the very maximum time frame in which P3HT based OFETs can be used is about 20 days as upper limit. A protocol for improving their durability was then applied. As a proof-of-concept, agarose gel was employed as protecting layer for the P3HT channel when the device was not in use. In the proposed set up, 1%_w_ agarose (300 μL) solution was poured on the P3HT film and let jellify directly in the measuring well. Then, the devices could be stocked in the dark at ambient conditions. The important point is that the device was constantly kept in contact with the water trapped in the gel-matrix. When needed, the agarose film could be easily removed and substituted with water for further device electrical characterization. After use, the gel layer could be deposited again. Shelf life was evaluated in terms of I_D_ measured on different days by acquiring a transfer curve, as reported in Figure 10. It can be seen that currents fluctuate in the order of the microampere, thus suggesting that agarose can be suitable for this application. More extensive studies will be performed to consider alternative protecting agents and/or strategies to further extend EGOFET durability.

## Conclusions

The investigation of the degradation processes occurring on P3HT film serving as semiconducting channel material in water-gated field-effect-transistors was carried out adopting the measurement protocols successfully set in single-molecule biosensing experiments. A benchmark polymer without any additional treatment was used in the development of water-gated FETs to study its reliability on prolonged use. It was observed that no hysteresis and limited leakage currents were achieved not only by operating the OS in a voltage range where typical parasitic electrochemical processes are absent (i.e., water electrolysis, polymer oxidation), but also by the suitable set up of the measuring protocol. Furthermore, it was shown that water incubation more than operation in cycling mode is responsible for the initial abrupt current loss occurring in the first 20 h. Such behavior accounts for the unavoidable presence of water in between the polymer chains. The role of degradation in water for long period was also highlighted by SEM, AFM, and XPS analyses demonstrating that roughness increases and additional hydroxyl moieties are present. A model of P3HT EGOFET degradation operating the device over 1 week continuously was proposed suggesting that the current loss is due to the V_T_ shift by a bi-exponential decay. On the other hand, P3HT based devices could keep their electrical performance over a prolonged period if stored under ambient conditions with a protecting layer of agarose gel. Though we are aware that particular conditions and materials were applied in this study, this work can be a guide to investigate aspects of EGOFET device stability which could be critical for their use in transistor biosensors.

## Data Availability Statement

All datasets generated for this study are included in the manuscript/Supplementary Files.

## Author Contributions

RP, KM, EM, and AT carried out the EGOFET fabrication, including measuring cell design, and the electrical characterization. RP wrote the first draft of the manuscript and performed XPS characterization. KM set up the measuring protocol. CD and GS were responsible for interdigitated electrode fabrication, SEM, and AFM analyses. NC contributed to the investigation of spurious electrochemical processes. LT supervised the overall study, coordinated the team, and revised the manuscript as approved by all the authors.

### Conflict of Interest

The authors declare that the research was conducted in the absence of any commercial or financial relationships that could be construed as a potential conflict of interest.
