# Supplementary material for: A Study on the Stability of Water-Gated Organic Field-Effect-Transistors Based on a Commercial p-Type Polymer
Source: Front Chem. 2019 Oct 10;7:667. doi: 10.3389/fchem.2019.00667 (PMC6795764; doi:10.3389/fchem.2019.00667)
Supplement: Supplementary file 1 [file Data_Sheet_1.PDF]

Supplementary Material

A study on the stability of water-gated organic field-effect-transistors based on a commercial p-type polymer

Rosaria Anna Picca[1,2*], Kyriaki Manoli[1,2], Eleonora Macchia[1,3], Angelo Tricase[1], Cinzia Di Franco[4], Gaetano Scamarcio[2,4,5], Nicola Cioffi[1,2], Luisa Torsi[1,2,3*]

[1]Dipartimento di Chimica - Università degli Studi di Bari “Aldo Moro”, Bari, Italy

[2]CSGI (Consorzio per lo Sviluppo dei Sistemi a Grande Interfase), Unità di Bari, Bari, Italy

[3]Center for Functional materials, The Faculty of Science and Engineering, Åbo Akademi University, Turku, Finland

[4]CNR - Istituto di Fotonica e Nanotecnologie, Unità di Bari, Bari, Italy

[5]Dipartimento Interateneo di Fisica “M. Merlin” - Università degli Studi di Bari “Aldo Moro”, Bari, Italy

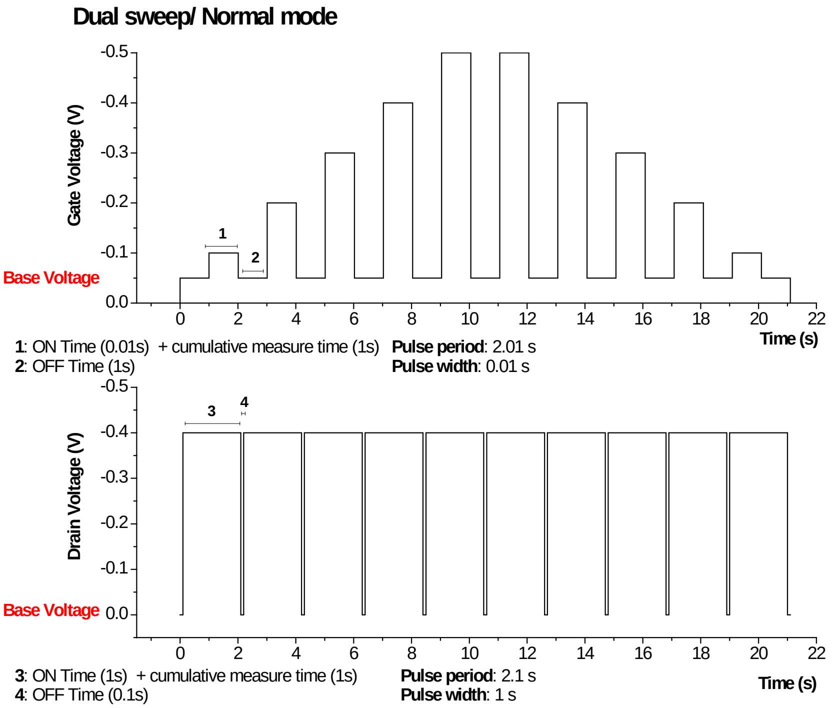

Figure S1. Plot of the duty-cycle employed in this study, showing the pulsed mode applied on G in the upper panel and the corresponding pulsed duty-cycle applied to D in the lower panel.
